# Central but Not Systemic Administration of Ghrelin Induces Wakefulness in Mice

**DOI:** 10.1371/journal.pone.0041172

**Published:** 2012-07-17

**Authors:** Éva Szentirmai

**Affiliations:** 1 Washington, Wyoming, Alaska, Montana and Idaho (WWAMI) Medical Education Program, Washington State University, Spokane, Washington, United States of America; 2 Department of Veterinary and Comparative Anatomy, Pharmacology and Physiology, Washington State University, Spokane, Washington, United States of America; 3 Sleep and Performance Research Center, Washington State University, Spokane, Washington, United States of America; Simon Fraser University, Canada

## Abstract

Ghrelin is a brain-gut peptide hormone widely known for its orexigenic and growth hormone-releasing activities. Findings from our and other laboratories indicate a role of ghrelin in sleep regulation. The effects of exogenous ghrelin on sleep-wake activity in mice are, however, unknown. The aim of the present study was to determine the sleep-modulating effects of ghrelin after central and systemic administrations in mice. Sleep-wake activity after intracerebroventricular (icv) administration of 0.2, 1 and 5 µg ghrelin and intraperitoneal injections of 40, 100, and 400 µg/kg ghrelin prior to light onset were determined in C57BL/6 mice. In addition, body temperature, motor activity and 1-hour food intake was measured after the systemic injections. Sleep effects of systemic ghrelin (40 and 400 µg/kg) injected before dark onset were also determined. Icv injection of ghrelin increased wakefulness and suppressed non-rapid-eye-movement sleep and electroencephalographic slow-wave activity in the first hour after injections. Rapid-eye-movement sleep was decreased for 2–4 hours after each dose of ghrelin. Sytemic administration of ghrelin did not induce changes in sleep-wake activity in mice at dark or light onset. Motor activity and body temperature remained unaltered and food intake was significantly increased after systemic injections of ghrelin given prior the light period. These findings indicate that the activation of central, but not peripheral, ghrelin-sensitive mechanisms elicits arousal in mice. The results are consistent with the hypothesis that the activation of the hypothalamic neuronal circuit formed by ghrelin, orexin, and neuropeptide Y neurons triggers behavioral sequence characterized by increased wakefulness, motor activity and feeding in nocturnal rodents.

## Introduction

Ghrelin is a brain-gut peptide hormone widely known for its orexigenic and growth hormone-releasing activities [1]. In peripheral organs, the main source of ghrelin is the stomach but it is also synthesized in the small intestine, pancreas, heart, kidney, and the gonads (reviewed in [2]). In the brain, ghrelin is produced by hypothalamic neurons in the arcuate nucleus (ARC), lateral hypothalamus (LH), paraventricular nucleus (PVN) and by a distinct population of neurons in the area surrounded by the third ventricle, PVN, ventromedial hypothalamic nucleus (VMH), dorsomedial hypothalamic nucleus and ARC [3].

Two forms of circulating ghrelin exist [4]; a non-acylated form (des-acyl ghrelin) and the form which is posttranslationally acylated by ghrelin-O-acyltransferase [5]. The latter is considered the biologically active form and is usually referred to as ghrelin in the literature. Ghrelin binds to a G protein-coupled receptor, known as the growth hormone secretagogue receptor 1a (GHS-R1a) [1]. This receptor is widely expressed in various brain regions, including hypothalamic nuclei such as the ARC, suprachiasmatic nucleus (SCN), LH, and VMH, and extrahypothalamic areas such as the pituitary, dorsal vagal complex, hippocampus, substantia nigra, ventral tegmental area and nucleus accumbens [6]–[9]. In peripheral organs, ghrelin receptor is present in the pancreas, spleen, myocardium, adipose tissue, thyroid, and adrenal gland [10] and also expressed on vagal afferents [11].

Administration of ghrelin facilitates gastric acid secretion and gastric emptying, stimulates food intake, decreases energy expenditure and increases adiposity [12]–[14]. Circulating ghrelin levels are elevated in the inter-digestive periods and are suppressed postprandially [15]. Furthermore, ghrelin has been implicated in glucose homeostasis, neuroprotection, cell proliferation, immune, reproductive and cardiovascular functions, learning and memory (reviewed in [2], [16]–[17]. Ghrelin plays a significant role in gut-brain communication. The food intake stimulating effects of ghrelin are mediated by feeding regulatory hypothalamic centers [18]. Additionally, some [11], [19]–[21] but not all [22], studies suggest that feeding-stimulatory signals via the vagus nerve are required for orexigenic actions of systemic ghrelin.

**Figure 1:**
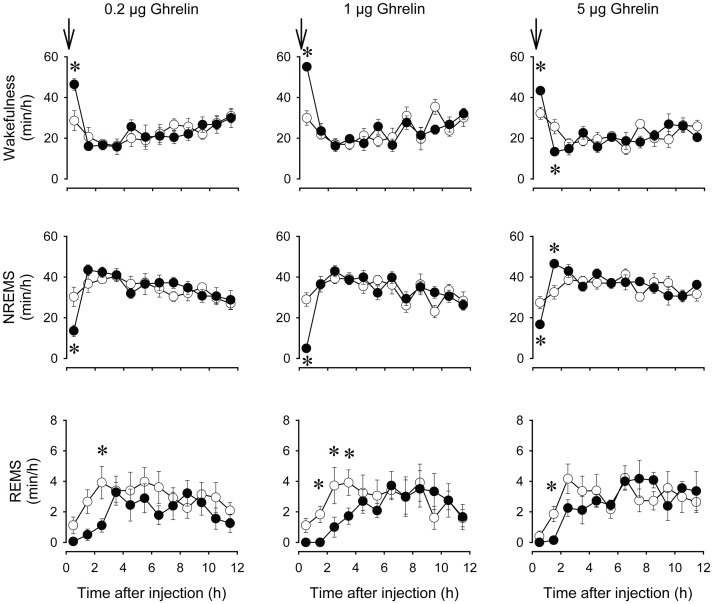
Wakefulness, non-rapid-eye-movement sleep (NREMS), and rapid-eye-movement sleep (REMS) after intracerebroventricular (icv) administration of 0.2, 1 and 5 µg ghrelin at light onset in mice. Open symbols: isotonic saline injection, solid symbols: ghrelin injection. Data were calculated in 1-h time blocks. Arrow: time of the injections. Asterisks denote significant differences between baseline and treatment days (post hoc paired t-test).

Findings from our and other laboratories indicate a role of ghrelin in sleep regulation. Sleep deprivation increases plasma and hypothalamic ghrelin levels in rats [23]. Systemic, intracerebroventricular (icv) and hypothalamic microinjections of ghrelin stimulate wakefulness, suppress non-rapid-eye-movement sleep (NREMS) and rapid-eye-movement sleep (REMS) in rats [24]–[26]. Ghrelin receptor knockout mice (originally named as GHS-R1a −/− mice [27]) lack or have attenuated arousal responses to wake-promoting stimuli such as fasting and exposure to novel environment [28]. Preproghrelin knockout mice (ghrelin −/− mice [29]) display severe sleep and thermoregulatory impairments when challenged with fasting in a cold environment [30]. They also have slightly altered sleep-wake activity at thermoneutral ambient temperature [31] but mount normal food anticipatory responses to food restriction [32]. In humans, hexarelin, the most potent synthetic agonist of the ghrelin receptors, suppresses stage 4 sleep, while the effects of ghrelin itself is variable depending on the age, gender and time of administration (reviewed in [33]).

**Figure 2:**
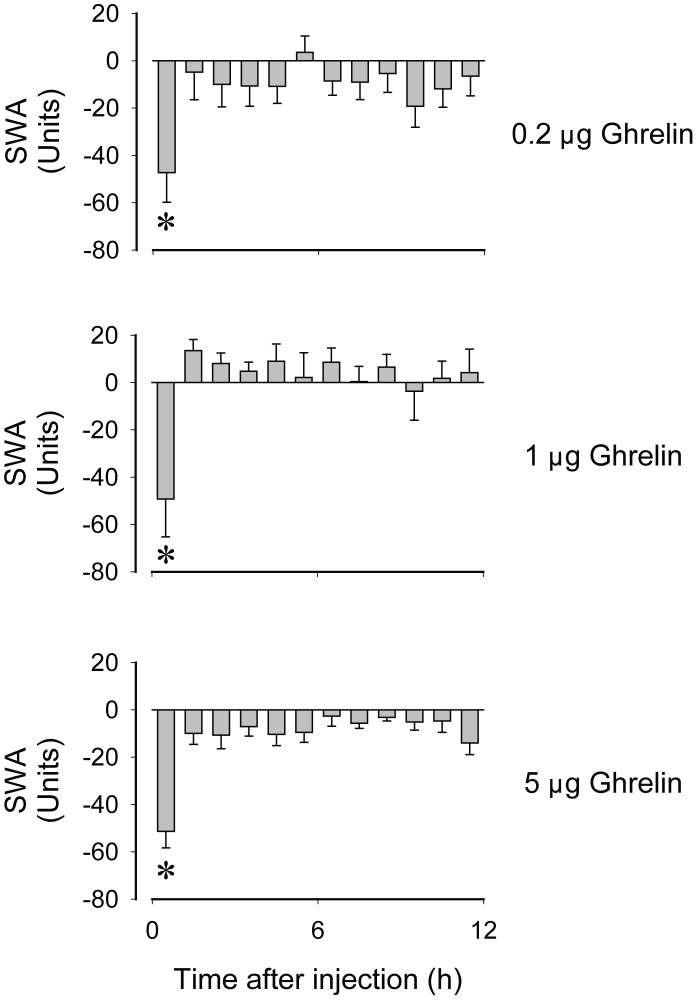
Changes in slow-wave activity (SWA) of the encephalogram after icv administration of 0.2, 1 and 5 µg ghrelin at light onset in mice. Data are expressed as difference from baseline in 1-h time blocks. Arrow: time of the injections. Asterisks denote significant differences between baseline and treatment days (post hoc paired t-test).

The availability of genetic mutant strains made mice an increasingly favored animal model for research to gain a better understanding the signaling mechanisms in sleep regulation. Sleep of mouse strains with loss-of-function mutation of the ghrelin signaling system is well characterized but the effects of exogenous ghrelin on sleep-wake activity in this species are poorly understood. In the present study, we determined the effects of ghrelin on sleep-wake activity after central and systemic administrations in mice. We report that icv injections of ghrelin increase wakefulness and suppress sleep, while systemic administration of the peptide fails to alter sleep-wake activity in mice.

## Materials and Methods

### Animals and Surgery

Male, 4–6 months old C57BL/6 mice were used in the experiments. The animals were housed individually in temperature-controlled, sound-attenuated environmental chambers at 30±1°C ambient temperature (thermoneutral for mice) with controlled lighting (LD 12∶12 h). Water and food were available *ad libitum* throughout the experiments. All animal work was conducted in accordance with the recommendations in the Guide for the Care and Use of Laboratory Animals of the National Institutes of Health. The protocol was approved by the Committee on the Ethics of Animal Experiments of the Washington State University (Permit Number: 3948). All surgeries were performed using ketamine-xylasine anesthesia [87 and 13 mg/kg intraperitoneally (ip), respectively], and all efforts were made to minimize pain. Thirty mice were used in the experiments, all were implanted with cortical electroencephalographic (EEG) electrodes and electromyographic (EMG) electrodes placed in the dorsal neck muscles for sleep recordings. The EEG and EMG electrodes were connected to a pedestal, which was fixed to the skull with dental cement. For icv injections, 6 mice were implanted with an additional guide cannula (22 GA: Plastics One, Roanoke, VA) targeting the lateral ventricle (coordinates of the tip of the cannula: 0.46 mm posterior and 1 mm lateral to bregma and 2.5 mm ventral to the surface of the skull; [34]). For core body temperature and locomotor activity recordings, 12 mice were implanted with telemetry transmitter in the abdominal cavity. Mice were allowed to recover from surgery for at least 2 weeks before any experimental manipulations.

**Figure 3:**
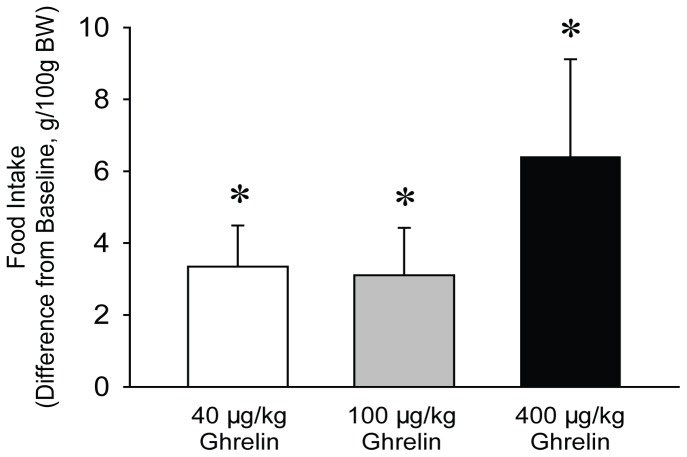
Changes in food intake in the first h after systemic injection of 40, 100 and 400 µg/kg ghrelin at light onset in mice. Data are expressed as difference from baseline. Asterisks denote significant differences between control and treatment days (post hoc paired t-test).

### Experimental Procedures

#### Experiment 1: The effects of intracerebroventricular injection of ghrelin on sleep-wake activity

After recovery from surgery, mice were handled daily to habituate them to the experimental procedures. On the baseline day, the animals were injected with isotonic saline (icv, 2 µl/mouse). On the test day, rat/mouse ghrelin (Bachem, Torrance, CA; dissolved in isotonic saline) was injected icv in the same volume. The order of the baseline and test days was counterbalanced. Three doses of ghrelin, 0.2, 1 and 5 µg were tested on the same group of mice; for each dose, separate baseline days were recorded. The ghrelin treatments were at least 7 days apart. All injections were performed 10–15 min prior to the onset of the light phase. Recordings were made for 12 h after the injections.

#### Experiment 2: The effects of systemic injection of ghrelin on sleep-wake activity, body temperature, locomotor activity and food intake

Twelve mice were habituated to daily ip injections during the recovery period from surgery. On the baseline day, the animals were injected with isotonic saline (ip, 0.1 ml/10 g body weight). On the experimental day, ghrelin was administered ip in the same volume. Three doses of ghrelin, 40, 100 and 400 µg/kg injected before light onset, were tested on the same group of mice; for each dose, separate baseline days were recorded. Sleep-wake activity, body temperature and motor activity were recorded for 6 h after the treatment. Food intake in the first hour after the saline and ghrelin treatments was also measured. The animals received pre-weighed food pellets in their cage immediately after the injections. The leftovers were collected and measured 1 h later. In a separate group of mice (n = 12), the effects of 40 and 400 µg/kg ghrelin injected prior to dark onset were tested on sleep-wake activity. EEG and EMG were recorded for 6 h after the injections; no other measurements were taken from this group of animals. The order of the saline and ghrelin days for both groups of animals was counterbalanced and the ghrelin treatments were separated by at least 7 days. All injections were performed 5–10 min prior the onset of the dark or light phases.

### Sleep-wake Activity Recordings

Seven to ten days after the surgery, animals were connected to recording cables, which were further routed to a Grass Model 15 Neurodata amplifier system (Grass Instrument Division of Astro-Med, Inc., West Warwick, RI). The EEG signals were filtered below 0.5 and above 30.0 Hz. The EMG signals were filtered with low and high cutoff frequencies at 100 and 10,000 Hz, respectively. The outputs from the amplifiers were fed into an analog-digital converter (digitized at 256 Hz) and collected by computer (SleepWave software, Biosoft Studio, Hersey, PA). Sleep-wake states were scored visually off-line in 10-s segments according to the following, conventional criteria. NREMS: high-voltage EEG delta waves (0.5–4 Hz) and decreased muscle tone; REMS: predominant EEG theta activity (6–8 Hz) and lack of muscle tone with occasional muscle twitches; wakefulness: low-voltage EEG activity, and varying levels of increased muscle activities. Time spent in wakefulness, NREMS and REMS was calculated in 1- and 6-h blocks. In experiment 1, EEG power data from each artifact free 10-s segment were subjected to off-line spectral analysis by fast Fourier transformation and EEG power in the range of 0.5 to 4.0 Hz during NREMS was used to compute EEG slow-wave activity (SWA). EEG SWA data were normalized for each animal by using the average EEG SWA across 12 h on the baseline day as 100. The results were averaged in 1-h bins.

### Telemetry Recordings

Core body temperature and locomotor activity were recorded by Mini Mitter telemetry system (Philips Respironics, Bend, OR) in Experiment 2 after light onset injections. Temperature and activity values were collected every 1 and 10 min, respectively, throughout the baseline and treatment days and were averaged into 1- and 6-h time blocks.

### Statistics

NREMS, REMS, wakefulness, SWA, body temperature and motor activity data were calculated in 1-h blocks. Two-way ANOVA for repeated measures was performed on wakefulness, NREMS, REMS, SWA, body temperature and motor activity for each dose of ghrelin across the 6- or 12-h recording period. When ANOVA indicated significant effects, post hoc paired t-tests were performed for all measurements. Food intake was analyzed by paired t-test.

## Results

### Experiment 1: The Effects of Intracerebroventricular Injection of Ghrelin on Sleep-wake Activity

Icv injections of ghrelin at light onset induced dose-dependent increases in wakefulness with the concomitant suppression of NREMS and REMS in the first hour (Figure 1 and Table 1). The lowest dose significantly increased wakefulness and suppressed NREMS in the first post-injection hour as indicated by the post hoc t-test. REMS was suppressed for three hours after the injection but post hoc analysis showed that this effect of ghrelin reached statistical significance in the third hour. The 1 µg dose elicited similar changes in sleep architecture. Wakefulness was increased and NREMS was suppressed by ∼84% in the first hour after the injection. The amount of REMS was below baseline for 4 hours after ghrelin treatment with the complete disappearance of REMS in the first 2 hours. Post hoc analysis revealed significant suppression in REMS for hours 2–4. Administration of 5 µg ghrelin elicited biphasic changes in sleep-wake activity. In the first hour after injection wakefulness was increased and NREMS was decreased significantly. In the second hour, however, wakefulness was suppressed and NREMS was elevated compared to baseline. Time in REMS was also suppressed by 5 µg ghrelin with a significant effect confined to the second hour post-injection. EEG SWA was significantly suppressed in the first post-injection hour after each dose of ghrelin (Figure 2 and Table 1).

**Table 1:** The effects of intracerebroventricular administration of 0.2, 1 and 5 µg ghrelin on wakefulness, non-rapid-eye-movement sleep (NREMS), rapid-eye-movement sleep (REMS) and slow-wave activity of the electroencephalogram (SWA) in mice: statistical results.

	0.2 µg ghrelin			1 µg ghrelin			5 µg ghrelin		
	df	F	p	df	F	p	df	F	p
***WAKE***									
Treatm.	1,5	7.69	P<0.05	1,5	17.2	P<0.05	1,5	0.3	n.s.
Time	5,25	14.2	P<0.05	5,25	32.0	P<0.05	5,25	27.0	P<0.05
Treatm. × Time	5,25	2.1	n.s.	5,25	5.9	P<0.05	5,25	4.1	P<0.05
***NREMS***									
Treatm.	1,5	1.9	n.s.	1,5	8.5	P<0.05	1,5	1.2	n.s.
Time	5,25	13.4	P<0.05	5,25	34.0	P<0.05	5,25	23.8	P<0.05
Treatm. × Time	5,25	2.9	P<0.05	5,25	6.2	P<0.05	5,25	5.7	P<0.05
***REMS***									
Treatm.	1,5	6.7	P<0.05	1,5	11.6	P<0.05	1,5	3.9	n.s.
Time	5,25	5.8	P<0.05	5,25	5.2	P<0.05	5,25	8.8	n.s.
Treatm. × Time	5,25	1.2	n.s.	5,25	2.4	n.s.	5,25	1.0	n.s.
***SWA***									
Treatm.	1,5	2.9	n.s.	1,5	0.1	n.s.	1,5	25.1	P<0.05
Time	5,25	3.8	P<0.05	5,25	6.1	P<0.05	5,25	12.5	P<0.05
Treatm. × Time	5,25	8.6	P<0.05	5,25	11.3	P<0.05	5,25	15.4	P<0.05

Treatm.: Treatment; NREMS: non-rapid-eye-movement sleep; REMS: rapid-eye-movement sleep; n.s.: not significant, df: degrees of freedom.

### Experiment 2: The Effects of Systemic Injection of Ghrelin on Sleep-wake Activity, Body Temperature, Locomotor Activity and Food Intake

None of the tested doses of ghrelin had significant effects on sleep-wake activity after systemic injection at dark or light onset (Tables 2, 3, 4 and 5). Light onset injection of ghrelin did not affect body temperature or motor activity (Table 2) but significantly increased feeding in the first hour (Figure 3).

**Table 2:** The amount of wakefulness (min), NREMS (min), REMS (min), activity counts and body temperature (°C) after systemic, light onset administration of ghrelin.

		Saline –1 h	Ghrelin –1 h	Saline –6 h	Ghrelin –6 h
**40 µg/kg**	WAKE	29.3±4.1	23.6±0.9	100.9±8.8	96.6±5.4
	NREMS	28.8±3.6	34.0±0.8	229.3±7.4	233.2±5.8
	REMS	1.9±0.6	2.4±0.6	29.8±2.0	30.3±3.2
	Activity	845.2±129.3	763.3±161.2	2532.5±419.5	2303.2±412.3
	BodyTemp.	36.7±0.3	36.8±0.2	36.1±0.2	36.2±0.2
**100 µg/kg**	WAKE	23.3±3.0	22.7±2.1	94.7±4.9	91.0±5.7
	NREMS	34.3±2.9	35.0±2.0	234.0±5.3	237.7±5.6
	REMS	2.4±0.4	2.3±0.3	31.2±2.6	31.3±1.9
	Activity	688.8±127.5	646.8±105.1	2155.3±265.6	2224.1±299.6
	BodyTemp.	36.7±0.1	36.6±0.2	36.2±0.1	36.1±0.1
**400 µg/kg**	WAKE	24.5±1.3	21.5±1.3	95.0±3.9	95.5±3.5
	NREMS	33.6±1.1	36.4±1.4	235.5±4.4	236.2±3.8
	REMS	1.9±0.3	2.1±0.3	29.5±1.8	28.3±1.9
	Activity	863.8±92.0	685.3±107.2	2444.8±243.6	2247.3±227.6
	BodyTemp.	36.7±0.1	36.5±0.1	36.1±0.1	36.1±0.1

**Table 3:** The effects of systemic, light onset administration of 40, 100 and 40 µg/kg ghrelin on wakefulness, sleep, motor activity and body temperature in mice: statistical results.

	40 µg/kg ghrelin			100 µg/kg ghrelin			400 µg/kg ghrelin		
	df	F	p	df	F	p	df	F	p
***WAKE***									
Treatm.	1,5	0.6	n.s.	1,11	0.4	n.s.	1,11	0.0	n.s.
Time	5,25	16.7	P<0.05	5,55	14.7	P<0.05	5,55	18.4	P<0.05
Treatm. × Time	5,25	1.1	n.s.	5,55	0.2	n.s.	5,55	0.5	n.s.
***NREMS***									
Treatm.	1,5	1.5	n.s.	1,11	0.4	n.s.	1,11	0.0	n.s.
Time	5,25	10.9	P<0.05	5,55	8.3	P<0.05	5,55	14.4	P<0.05
Treatm. × Time	5,25	0.9	n.s.	5,55	0.2	n.s.	5,55	0.5	n.s.
***REMS***									
Treatm.	1,5	0.0	n.s.	1,11	0.0	n.s.	1,11	0.5	n.s.
Time	5,25	11.1	P<0.05	5,55	27.4	P<0.05	5,55	13.8	P<0.05
Treatm. × Time	5,25	1.4	n.s.	5,55	0.6	n.s.	5,55	0.7	n.s.
***Motor Activity***									
Treatm.	1,5	0.0	n.s.	1,11	0.0	n.s.	1,11	0.9	n.s.
Time	5,25	5.4	P<0.05	5,55	15.9	P<0.05	5,55	26.7	P<0.05
Treatm. × Time	5,25	7.0	P<0.05	5,55	0.8	n.s.	5,55	1.0	n.s.
***Body temperature***									
Treatm.	1,5	3.1	n.s.	1,11	4.2	n.s.	1,11	1.5	n.s.
Time	5,25	19.0	P<0.05	5,55	49.7	P<0.05	5,55	34.0	P<0.05
Treatm. × Time	5,25	0.2	n.s.	5,55	0.3	n.s.	5,55	1.7	n.s.

**Table 4:** The amount of wakefulness (min), NREMS (min), REMS (min) after systemic, dark onset administration of 40 and 400 µg/kg ghrelin.

		Saline –1 h	Ghrelin –1 h	Saline –6 h	Ghrelin –6 h
**40** **µg/kg**	WAKE	44.6±7.3	47.1±2.7	202.8±36.0	215.1±37.4
	NREMS	14.5±2.7	12.1±2.5	99.2±20.1	88.4±17.6
	REMS	0.9±0.7	0.9±0.4	6.6±1.9	5.0±1.5
**400** **µg/kg**	WAKE	44.0±1.8	40.9±1.6	240.0±12.1	245.7±11.1
	NREMS	15.9±1.7	18.9±1.5	116.2±11.3	110.1±10.1
	REMS	0.1±0.1	0.2±0.2	3.8±1.1	4.2±1.1

**Table 5:** The effects of systemic, dark onset administration of 40 and 40 µg/kg ghrelin on wakefulness and sleep in mice: statistical results.

	40 µg/kg ghrelin			400 µg/kg ghrelin		
	df	F	p	df	F	p
***WAKE***						
Treatm.	1,5	5.3	n.s.	1,11	0.5	n.s.
Time	5,25	3.3	P<0.05	5,55	1.0	n.s.
Treatm. × Time	5,25	1.2	n.s.	5,55	1.5	n.s.
***NREMS***						
Treatm.	1,5	4.7	n.s.	1,11	0.7	n.s.
Time	5,25	3.6	P<0.05	5,55	1.0	n.s.
Treatm. × Time	5,25	1.2	n.s.	5,55	1.6	n.s.
***REMS***						
Treatm.	1,5	5.3	n.s.	1,11	0.6	n.s.
Time	5,25	2.5	n.s.	5,55	2.5	P<0.05
Treatm. × Time	5,25	1.2	n.s.	5,55	1.1	n.s.

## Discussion

The major findings of the present study are that 1) icv injection of ghrelin increases wakefulness, suppresses NREMS, REMS and EEG SWA in mice, 2) systemic administration of ghrelin stimulates feeding but fails to alter sleep-wake activity. Collectively, these data extend our knowledge about ghrelin’s role in sleep-wake regulation by differentiating between its central and peripheral effects, and by showing its wake-promoting action after central administration in a new species. Our data also confirm the feeding-stimulating effect of systemic ghrelin injections [1], [12]–[14].

We previously showed that icv injection of ghrelin at light onset dose-dependently increases wakefulness and suppresses NREMS and REMS in rats [25]. Consistent with ghrelin’s wake-promoting effect, icv, intra-ventral tegmental area, intra-laterodorsal tegmental area injections of ghrelin increase motor activity [35]–[38]. The mechanism of central administration of ghrelin’s wake-promoting effect is not completely understood. Ghrelin receptors are widely expressed in the hypothalamus including the ARC, PVN, SCN, and the LH as well as in various extrahypothalamic structures [6]–[9]. Our previous studies revealed that local microinjections of ghrelin into the LH, medial preoptic area (MPA) or PVN of the hypothalamus induces dose-dependent increases in wakefulness, food intake and suppresses NREMS, REMS in rats [26]. Interestingly, icv or lateral hypothalamic injections of neuropeptide Y (NPY) [39] and orexin [40] have almost identical wake-promoting and feeding-stimulating effects in rats. In the hypothalamus, ghrelin-, orexin- and NPY-producing neurons form a well-characterized network. Ghrelin-producing neurons are located in the ARC, LH, PVN, and in the hypothalamic area adjacent to the ARC, VMH, dorsomedial hypothalamic nucleus and PVN [3]. There are reciprocal connections between hypothalamic ghrelin-, orexin- and NPY-ergic neurons. Orexin is well-known about its role in maintaining wakefulness [41]. Icv and LH injections of NPY increase wakefulness in rats [39]. It is possible that the wake-promoting effects of ghrelin are due to their stimulatory actions on hypothalamic orexin and NPY-ergic neurons.

Icv ghrelin injections suppressed EEG SWA, the NREMS-associated EEG delta power, in the first hour. This measurement of the EEG is commonly used as an indication of NREMS intensity [42]. We observed similar suppression of EEG SWA after microinjections of ghrelin into the LH, PVN or MPA in rats [26]. The animals had significantly reduced NREMS time in the first hour post-injection both in the present study and after hypothalamic microinjections. It is possible that shortening of NREMS interferes with the mechanism responsible for increasing NREMS intensity which explains the suppressed EEG power in the slow (delta) wave range.

The effect of systemic administration of ghrelin or ghrelin receptor agonists on sleep-wake activity is less consistent in the literature. When ghrelin is administered in intravenous (iv) pulses during the first part of the night, it induces slight increases in sleep in young men [43] but when it is administered during the second part of the night, it lacks its modest sleep-promoting activity [44]. In healthy women, young or elder, ghrelin has no effect on sleep [45]–[46]. Pulsatile iv administration of growth-hormone-releasing peptide-6 does not affect total sleep time but modestly increases stage 2 sleep [47]. MK-677, an orally active ghrelin receptor agonist, increases the duration of stage 4 sleep by 50% and REMS by 20% in young male subjects after one week of treatment [48]. Iv bolus injection of growth hormone releasing peptide-2 does not have any effect on sleep EEG or on the amount of slow-wave sleep [49]. Pulsatile, iv injection of hexarelin to young males decreases deep, stage 4 sleep during the first half of the night and suppresses EEG delta power during NREMS [50]. In rats, sequential iv injections of 10 µg/rat ghrelin increased wakefulness and decreased NREMS and REMS for 30 min immediately after the injections [24]. The different species, injection schedule or the route of systemic administration may explain the lack of sleep effect of ip administered ghrelin in the present experiment.

In mice, one single paper reports increased sleep after ip, dark onset administration of 400 µg/kg ghrelin [51]. In our hands, the same dose of ghrelin did not induce sleep when injected before the dark or the light phase. An apparent difference between the two studies is the sleep-wake activity of the animals on the baseline day. In the study by Obál et al., mice slept less than 10% in the first hour of the baseline which increased to ∼22% after ghrelin injection. In our experiment, the amount of sleep was 25% on the baseline day which did not change significantly after ghrelin treatment. Differences in baseline sleep amounts likely reflect differences in the degree of habituation of the animals to the experimental procedure. In our experiments, we carefully habituated the animals to the ip injections for at least 7 days prior to the experiments to minimize stress, thus minimize the non-specific sleep suppressing effect of the injection procedure. It is possible, that the sleep-promoting effect of 400 µg/kg ghrelin injection reported by Obál et al is the reflection of the animals’ increasing habituation to the injection procedure. Nevertheless, we tested biologically effective doses of ghrelin as indicated by significantly increased feeding after each doses of systemic injection which confirms previous findings from other laboratories [12]–[14].

Regular, periodic feeding restricted to a few hours during the light period triggers food-anticipatory activity which is characterized by increased behavioral activity and wakefulness 1–4 h before the scheduled feeding time (reviewed in [32]). There is some controversy concerning the role that ghrelin might play in the control of food-anticipatory activity in mice. Food-anticipatory activity in ghrelin receptor knockout mice has been reported to be reduced in amount or shortened in duration in two studies [52], [53], while other studies, including ours, using receptor or ligand knockout mice did not confirm these effects [32], [54]. If peripheral sources of ghrelin drive or modulate the level of food-anticipatory activity in mice, then it is expected that systemic administration of exogenous ghrelin would also stimulate waking and behavioral activity. Our results provide no evidence for this thus further detract from the hypothesis that food-anticipatory behavior is regulated by gastric ghrelin.

The lack of wakefulness-modulating effect of systemic ghrelin injections suggest that circulating ghrelin does not activate the same central wake-promoting mechanisms that is stimulated by the brain-derived ghrelin and accessible to centrally injected peptide. A series of recent studies suggest that circulating ghrelin acts on peripheral targets to stimulate feeding, particularly, it was demonstrated that the gastric vagal afferent pathway also conveys orexigenic signals to the brainstem. For example, ip injection of ghrelin into vagotomized mice did not stimulate food intake [19], blockade of the gastric vagal afferent pathway abolished peripheral ghrelin-induced feeding [11], and bilateral midbrain transsection rostral to the NTS, or toxin-induced loss of neurons in the hindbrain that express dopamine b hydroxylase abolished ghrelin-induced feeding [21]. Our results suggest that activation of these peripheral mechanisms has no effect on sleep-wake activity in mice. The fact that systemic injections of ghrelin stimulated feeding but had no effect on sleep-wake activity indicate that the wake-promoting and feeding-stimulating actions of ghrelin are independent from each other and increased feeding by itself does not necessarily lead to prolonged wakefulness.

In conclusion, the current findings together with previous observations suggest that ghrelin elicits wakefulness and feeding when administered centrally prior to the rest, inactive phase in nocturnal rodents. These results are consistent with our hypothesis that the activation of the hypothalamic ghrelin – NPY – orexin neuronal circuit triggers behavioral sequence characterized by increased wakefulness, motor activity and feeding [26]. We named this set of behavior “dark onset syndrome” as it spontaneously occurs at the beginning of the dark or active period in nocturnal animals. Present results further support our hypothesis that ghrelin plays a fundamental role in triggering the behavioral sequence of dark onset syndrome.
